# Impact of early assessment and intervention by teams involving health and social care professionals in the emergency department: A systematic review

**DOI:** 10.1371/journal.pone.0220709

**Published:** 2019-07-31

**Authors:** Marica Cassarino, Katie Robinson, Rosie Quinn, Breda Naddy, Andrew O’Regan, Damien Ryan, Fiona Boland, Marie E. Ward, Rosa McNamara, Margaret O’Connor, Gerard McCarthy, Rose Galvin

**Affiliations:** 1 School of Allied Health, Faculty of Education and Health Sciences, Health Research Institute, Ageing Research Cluster, University of Limerick, Limerick, Ireland; 2 Emergency Department, Our Lady of Lourdes Hospital, Drogheda, Ireland; 3 Clinical Strategy and Programmes Division, Royal College of Surgeons in Ireland, Dublin, Ireland; 4 Graduate Entry Medical School, Faculty of Education and Health Sciences, University of Limerick, Limerick, Ireland; 5 Retrieval, Emergency and Disaster Medicine Research and Development Unit (REDSPoT), Emergency Department, University Hospital Limerick, Dooradoyle, Limerick, Ireland; 6 HRB Centre for Primary Care Research, Royal College of Surgeons in Ireland, Dublin, Ireland; 7 School of Psychology, Trinity College, the University of Dublin, Dublin, Ireland; 8 Emergency Department, St. Vincent’s University Hospital, Dublin, Ireland; 9 Department of Ageing and Therapeutics, University Hospital Limerick, Dooradoyle, Limerick, Ireland; 10 Emergency Department, Cork University Hospital, Cork, Ireland; University of South Australia, AUSTRALIA

## Abstract

**Background:**

Dedicated Health and Social Care Professional (HSCP) teams have been proposed for emergency departments (EDs) in an effort to improve patient and process outcomes. This systematic review synthesises the totality of evidence relating to the impact of early assessment and intervention by HSCP teams on quality, safety and effectiveness of care in the ED.

**Methods:**

A systematic literature search was conducted in April 2019 to identify experimental studies examining the effectiveness of ED-based HSCP teams providing services to adults aged ≥ 18 years old and including two or more of the following disciplines: occupational therapist, physiotherapist, medical social worker, clinical pharmacist, or speech and language therapist. Data extraction and quality appraisal of each study were conducted independently by two reviewers.

**Results:**

Six studies were included in the review (n = 273,886), all describing interdisciplinary Care Coordination Teams (CCTs) caring for adults aged ≥ 65 years old. CCT care was associated with on average 2% reduced rates of hospital admissions (three studies), improved referrals to community services for falls (one study), increased satisfaction (two studies) with the safety of discharge (patients and staff), and with the distribution of workload (staff), improved health-related quality of care (one study). No statistically significant differences between intervention and control groups emerged in terms of rates of ED re-visits, ranging between 0.2% and 3% (two studies); hospital length of stay (one hour difference noted in one study) or mortality rates (0.5% difference in one study). Increased rates of unplanned hospitalisations following the intervention (13.9% difference) were reported in one study. The methodological quality of the studies was mixed.

**Discussion:**

We found limited and heterogeneous evidence on the impact of HSCP teams in the ED, suggesting a reduction in hospital admissions as well as improved patient and staff satisfaction. More robust investigations including cost-effectiveness evaluations are needed.

## Introduction

Index visits to the emergency department (ED) are increasing at a rate that exceeds population growth [1]. As conceptualised within the Input-Throughput-Output model [2], high ED attendance is influenced by multiple factors internal and external to the acute care system which can present before, during and after ED admission. Extrinsic factors include population ageing and the associated increase in multimorbidity, organisational issues in primary care, patients’ subjective perceptions of illness gravity, healthcare services accessibility and quality, and lack of cost awareness [3]. On the other hand, finite hospital resources that are insufficient to meet patient demand often lead to slow patient flow and ED overcrowding, which in turn have been linked to negative patient and process outcomes [4–6]. While extrinsic factors relating to the increased incidence of ED attendance are complex and challenging to address, a number of quality improvement initiatives have been implemented in the ED to enhance patient flow, such as patient triage and streaming [3,7], although the extent of their effectiveness is still unclear [8]. Staffing in the ED has also been explored from several perspectives including resources, roles and scope of practice [9]. EDs have traditionally been staffed by doctors and nurses, where doctors were considered the key decision-makers in aspects of referral, admission, and discharge. Health and social care professionals (HSCPs) such as physiotherapists, occupational therapists, speech and language therapists, medical social workers and clinical pharmacists were called to the ED to consult on an ad hoc basis. Increasingly, these HSCPs have extended their scope of practice to work within the ED [10]: Physiotherapists offer timely management of ED patients with low urgency musculoskeletal conditions, contributing to enhance both ED cost-effectiveness as well as patient health outcomes [11,12]. Similarly, ED-based clinical pharmacists have a positive impact on quality, safety and cost-effectiveness of ED care by providing a range of services such as medication reconciliation and management [13]. By providing assessments of functional and social needs, occupational therapists and medical social workers working in the ED have reduced unnecessary hospital admissions particularly for older patients [14,15]. Some literature has also shown that speech and language therapists have been instrumental to improve screening procedures, such as swallow assessment, in the ED [16], although the evidence is still limited [10]. The representation of allied health professionals in the ED varies across studies and regions. In their review of HSCPs in the ED, Saxon et al [10] found mainly evidence on ED-based physiotherapists; a recent study found that physiotherapists, social workers and clinical pharmacists are the most common HSCPs (around 70%) in Australian EDs [17], whereas in the UK the types of allied health services in the ED vary based on the specific clinical demands [18]. More recently, there has been a growing body of primary research evidence supporting a more interdisciplinary approach to the management of patients in the ED [19–21]. To date, no systematic review has examined the totality of evidence relating to the impact of interdisciplinary ED teams that include HSCPs (working with or without traditional ED professionals such as doctors or nurses) on the quality, safety and cost-effectiveness of care. Furthermore, it is unclear whether specific target populations benefit more from such a model of care.

This systematic review aims to: 1) to explore the impact of early assessment or intervention conducted by interdisciplinary teams with two or more HSCP members in the ED on the quality, safety and cost-effectiveness of care of adults presenting to the ED; 2) to define the content of the assessment or intervention delivered by the HSCP team.

## Material and methods

### Protocol and registration

This review was conducted following the Preferred Reporting Items for Systematic Reviews and Meta-analyses (PRISMA) guidelines [22]. A details PRISMA checklist is included in S1 Appendix. The full review protocol is published elsewhere [23] and is registered with PROSPERO (CRD42018091794).

### Eligibility criteria

Studies were selected using the Population, Interventions, Comparators, Outcomes, and Study designs (PICOS) criteria, as follows:

- Population: Adults aged ≥18 years who present to the ED in need of care
- Intervention: Early assessment or interventions conducted in the ED by interdisciplinary teams comprising one or more HSCP members. Here, ‘early assessment and intervention’ refers to proactive assessment and intervention by the HSCP team following ED triage with/without assessment by a medical professional. Using the definition established by Naylor and colleagues [20,24], we defined “team” as an interdisciplinary group of two or more healthcare professionals who work collaboratively with patients to accomplish shared goals to achieve high quality care in the emergency department. Therefore, studies were included in the review only if the following criteria were met:
  ○ the interdisciplinary team included two or more of the following health and social care professionals: physiotherapist (PT); occupational therapist (OT); medical social worker (MSW); clinical pharmacist (CP); speech and language therapist (SLT); AND
  ○ the team operated within the ED (i.e., studies were excluded if patients were referred to a HSCP working in a team as secondary point of contact in a department other than the ED).
- Comparison: Usual care or another active intervention.
- Outcomes: The primary outcome of interest was ED length of stay (LOS). Secondary outcomes included: number of ED re-visits; rate of hospital admissions; patient and/or staff satisfaction; patient's health outcomes; morbidity; mortality; and cost-effectiveness.
- Study Design: The review included randomised controlled trials (RCTs), non-randomised controlled trials (nRCTs), controlled before-after studies (CBAs), interrupted time series (ITS) and repeated measures studies (RMS).

### Search

A comprehensive search string was developed by the authors and peer reviewed by the dedicated Education and Health Sciences (EHS) Faculty Librarian at the University of Limerick (Ireland) using the Peer Review of Electronic Search Strategies (PRESS) model[25]. Searches were carried by title and abstract in the following electronic databases from inception to April 2019: The Cumulative Index of Nursing and Allied Health Literature (CINAHL); Embase; the Cochrane Library; and MEDLINE. Detailed search strategies for each of the four databases have been included in the S2 Appendix. No restrictions in terms of language, date of publication or publication type were applied. The reference lists of included studies were also hand searched. All results were imported into the Rayyan citation management software[26], where duplicate citations were screened and removed.

### Study selection

A two-stage process was used to assess the results of the literature search. In stage 1, titles and abstracts were independently screened by two reviewers (MC and RG) against the inclusion criteria; in stage 2, the selected full-texts were screened by both reviewers to confirm inclusion in the final review. A comparison of included and excluded full-text studies was carried out by the two reviewers and discrepancies were resolved by consensus.

### Data collection process and data items

One reviewer (MC) extracted data from the included studies using a tailored data extraction form. The extraction form collected information relating to the authors and year of publication, setting, PICOS and duration of follow up. Information was extracted on all study outcomes and on the content of the assessment/intervention. A second reviewer (RG) independently verified the extracted outcomes and content of assessment/intervention; disagreements were resolved by consensus. A third author (KR) was designated to arbitrate disagreements where consensus could not be reached, but all disagreements were resolved by consensus.

### Risk of bias of individual studies

The quality of controlled studies (randomised, non-randomised, before-after) was critically appraised by two independent reviewers (MC and RG) using the Cochrane Collaboration’s Risk of Bias Tool [27] to assess for the following types of bias: selection, performance, detection, attrition, reporting, and other biases. The Cochrane Effective Practice and Organisation of Care (EPOC) risk of bias criteria [28] were employed to assess the risk of bias of interrupted time series and repeated measures studies. Disagreements between reviewers were resolved by consensus and a third reviewer (KR) resolved disagreements where necessary.

### Synthesis of results

A narrative synthesis was conducted by reporting the results of the included studies grouped by type of assessment/intervention and by outcome of interest. A meta-analysis was not possible due to the heterogeneity in study designs, health conditions, and outcomes reported. For this reason, related analyses, such as subgroup analyses and funnel plot assessment that were pre-planned were not carried out. We were also unable to evaluate the quality of evidence for outcomes using the GRADE methodology [29] due to the high heterogeneity in outcomes and thus the small number of studies exploring the same outcome (e.g., ED length of stay was explored just in one study).

## Results

### Study selection

**Fig 1:**
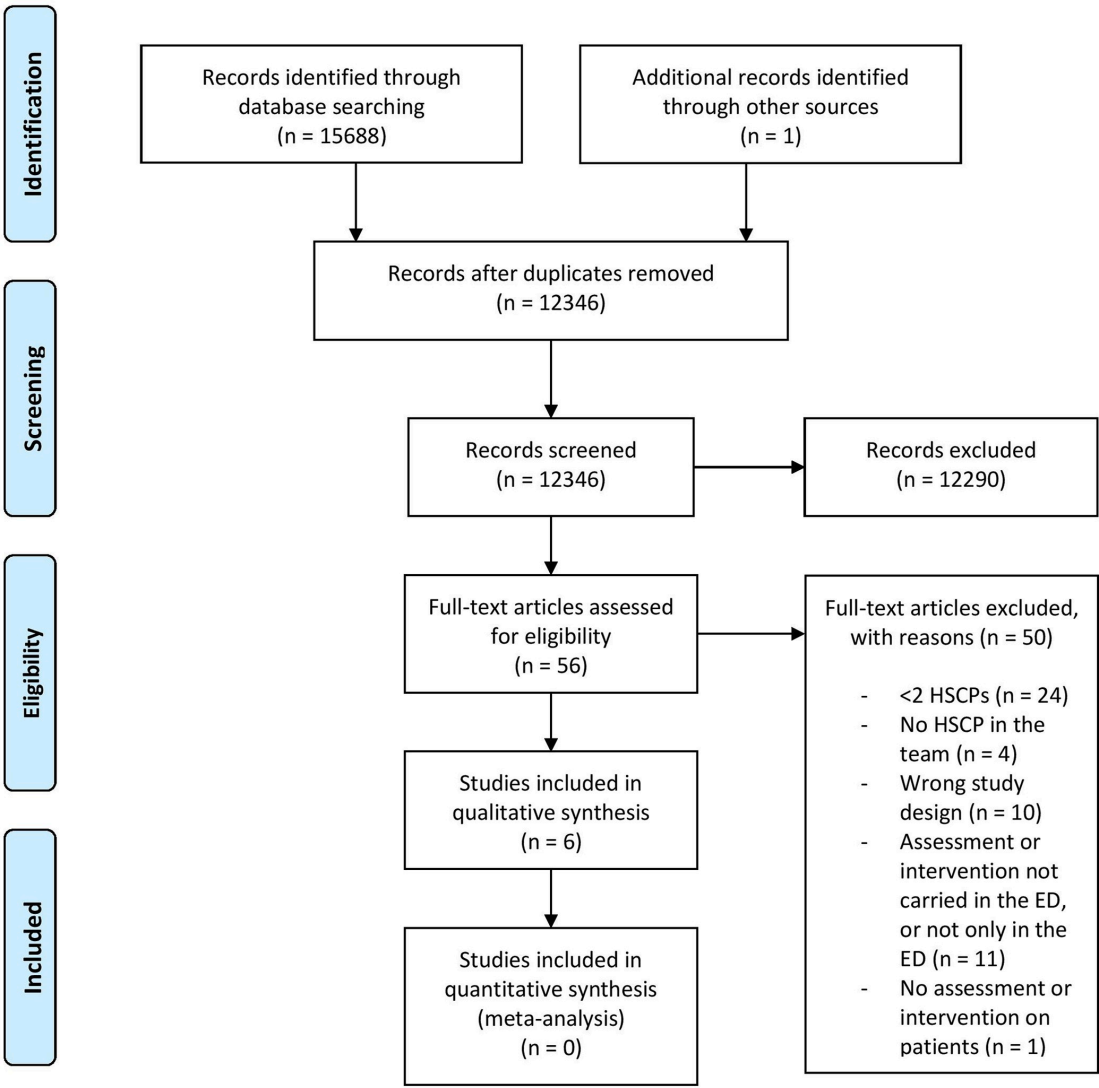
PRISMA flow of study selection.

Fig 1 describes the flow of studies in the review. A total of 15,689 records were retrieved. After excluding duplicates (n = 3,343), 12,346 titles and abstracts were screened, and 12290 were excluded because they did not meet the inclusion criteria (screening stage 1). A total of 56 full-text articles were then reviewed for inclusion (screening stage 2) and six of these were subsequently included by the two independent reviewers [1,30–34].

### Study characteristics

Detailed information about the included studies is reported in S1 Table in accordance with the PRISMA guidelines and the Template for Intervention Description and Replication (TIDieR) framework [35]. The study designs comprised of three nRCTs [1,30,31], one CBAs [34], and two RMSs [32,33]. All six studies were conducted in Australia and had a sample size ranging from 313 [34] to 175,400 [32], with a total of 273,886 patients involved (n = 143,942 as part of intervention groups). All the studies focused on adults ≥65 years whose health conditions varies across studies from falls only [34] to a variety of pre-specified index complaints [1,30,31,33]; one study did not specify the types of index conditions [32].

### Team composition and content of early assessment/intervention

All the studies described services provided by a care coordination team (CCT) comprising at least one OT, one PT and one MSW. In one study, SLTs and nurses were also stable members of the CCT [32], while in three studies SLTs, nurses and ED physicians were co-opted as needed [1,30,31]. In the three studies by Arendts and colleagues [1,30,31], at least one member of the CCT completed a comprehensive functional assessment (including falls risk, activities of daily living, cognition, and discharge needs) of the older patient to be incorporated into the medical decision about discharge to the community or hospital admission from the ED; in addition, CCT members provided specific services based on the needs that emerged during the assessment (content not specified), and coordinated implementation of post-discharge services in the community. Corbett et al. [32] described the CCT as mainly involved in case management and coordination of community services for older patients after discharge. Services were also provided to patients in the ED, but the content was not described. Similarly, in Moss et al. [33] the CCT provided a comprehensive discharge assessment and referral to internal or community-based service providers. Lastly, in Waldron et al. [34], the CCT used a newly introduced referral pathway that integrated an assessment of falls risk in the community for older patients presenting to the ED after a fall with a range of post-discharge multifactorial or single interventions (e.g., OT home visits, physical therapy).

### Effectiveness of assessment/intervention

None of the studies included in the review investigated ED length of stay or cost-effectiveness. Outcomes reported across the studies included incidence of hospital admission [1,32,33], hospital length of stay [31] following the ED index visit; rates of ED and/or hospital re-attendance [30,33]; quality of community referrals [34]; mortality [30]; patient and staff satisfaction [32,33]; health-related quality of life [32].

#### Hospital admission/length of stay

Three studies that described the implementation of ED-based CCTs [1,32,33] investigated rates of hospital admission from the ED. Two studies [1,32] found an approximate 2% reduced rate of hospital admission in the intervention groups as compared to usual care (n = 180,665). Specifically, Arendts et al. [1] noted significant lower odds of hospital admissions from the ED for older patients with musculoskeletal conditions (OR = 0.67, 95% CI = 0.49–0.93, p = 0.01) or with angina (OR = 0.71, 95% CI = 0.53–0.93, p = 0.01). Moss et al. [33] observed a 1.7% decrease in hospital admissions when comparing the year after introduction of the CCT with the year before (chi2 = 27.7, p<0.001). In addition, Arendts and colleagues [31] reported no significant differences in terms of hospital length of stay after admission from the ED between ED-based CCT assessment of older patients as compared to routine medical assessment (median length of stay: 88 hours in intervention vs. 87 in control, IRR = 0.97, p = 0.32). In contrast to the beneficial finding above, Arendts [30] found that there was a higher incidence of unplanned hospitalisation at one year in the CCT intervention group compared to controls (43.4% vs 29.5%, p<0.5, n = 2196).

#### ED or hospital re-attendance

Two studies examined differences in ED re-attendance across the groups [30,33]. Arendts et al. [30] reported that 17.9% of older patients undergoing CCT assessment and 14.8% of those in a matched control group re-attended the ED within 28 days (3% absolute difference with borderline statistical significance, p = 0.05); however, patients in the intervention group had higher rates of unplanned hospitalisations than the control group at one year follow-up (43.4% vs. 29.5%, p < 0.001). Moss et al. [33] found no significant changes in 12-month ED re-visits before and after introduction of a CCT to the ED (after: 3744, 8.6%; before: 3856, 8.8%; p = 0.28).

#### Community referrals

Moss et al. [33] reported that 81.5% of older adults seen by the ED-based CCT were discharged home, whereas 15.4% were admitted; however, the authors did not compare this result to a control group. Waldron and colleagues [34] noted a 17.2% increase in the number of referrals to community-based multifactorial interventions after the introduction of a new referral pathway conducted by the CCT team in the ED, as compared to a historical control group, and a 75% increase in quality of care as assessed by an external audit.

#### Mortality

Arendts [30] found similar mortality rates for older patients discharged from the ED following CCT assessment and a matched control group undergoing usual care at 28 days (1.3% vs 1.4%, p = 0.85) or one year follow-up (10.2% vs 10.7%, p = 0.66).

#### Patient reported outcomes

Patient satisfaction was assessed via questionnaire/survey in two studies [32,33], although only a small number of patients from the intervention group (n = 11 and n = 40 respectively) provided responses: Participants rated the CCT as helpful in offering safe discharge home, and would recommend it as a successful model of care. No patients cared for before the introduction of the CCT were assessed for this outcome. In addition, Corbett et al. [32] compared health-related quality of life in older adults before and 28 days after CCT assessment using the Assessment of Quality of Life (AQoL) questionnaire, and found small but significant improvements in terms of independent living (0.61 vs. 0.79, p = 0.04), social relationships (0.61 vs. 0.87, p = 0.009), physical senses (0.76 vs. 0.87, p = 0.04), psychological wellbeing (0.65 vs. 0.92, p = 0.003), and overall utility score (0.27 vs. 0.58, p = 0.006), but not in terms of reduction of illness (0.32 vs. 0.38, p = 0.14).

#### Staff satisfaction

Corbett [32] and Moss [33] investigated also ED staff’s level of satisfaction via survey or focus groups, and noted positive perceptions. In Corbett et al. [32] ED staff judged the interventions as promoting lessening of workload and higher effectiveness of the ED team. In Moss et al. [33], over 92% of the 68 ED staff members who completed a satisfaction survey rated the CCT as providing quality patient care, having a positive impact on patient discharge, being easily accessible, increasing staff morale, and worth recommending to other EDs.

### Risk of bias

Overall the risk of bias across the studies was mixed, as shown in Table 1. All six studies demonstrated a high risk of selection bias and unclear/high risk of performance bias due to the lack of randomisation or allocation concealment. The risk of detection bias was unclear for three studies where information about assessor’s blinding was missing and/or outcome measures were not objective (e.g., patient satisfaction). The two RMSs were deemed as having an unclear risk of attrition and reporting bias as well as a high risk of bias related to the absence of information about patients’ baseline characteristics.

**Table 1:** Risk of bias of included studies.

Domain	Arendts et al. (2012) nRCT [1]	Arendts et al. (2013) nRCT [30]	Arendts et al. (2013) nRCT [31]	Corbett et al. (2005) RMS [32]	Moss et al. (2002) RMS [33]	Waldron et al. (2011) CBA [34]
Random sequence generation	**-**	**-**	**-**	**-**	**-**	**-**
Allocation concealment	**-**	**-**	**-**	**-**	**-**	**-**
Performance bias	**-**	**-**	**-**	**?**	**?**	**-**
Detection bias	**+**	**+**	**?**	**?**	**?**	**+**
Attrition bias	**+**	**+**	**+**	**?**	**?**	**+**
Reporting bias	**+**	**+**	**+**	**?**	**?**	**+**
Other bias–baseline groups characteristics	**?**	**+**	**?**	**-**	**-**	**+**

Notes. Risk of bias presented as–(red) = high, ? (yellow) = unclear, + (green) = low for each domain. Controlled studies were assessed using the Cochrane Risk of Bias tool, while repeated measures studies were assessed in accordance with the Cochrane Effective Practice and Organisation of Care (EPOC) risk of bias criteria [28].

## Discussion

### Statement of principal findings

Our systematic review identified six studies (n = 273,886) describing early assessment and/or interventions conducted by ED-based CCTs involving HSCPs. Studies included were heterogeneous in nature with respect to study designs, index complaints, outcomes assessed and duration of follow up. No randomised controlled trials were included in our review. We found limited evidence to support the effectiveness of HSCP interventions in terms of significant reductions in rates of hospital admissions (three studies), increased patient and staff satisfaction (two studies), and improved integrated care (one study) for older adults. The changes observed due to the intervention, although significant, were very small. On the other hand, increased rates of unscheduled hospitalisations for the intervention than control group were reported in one study. No effects of the interventions emerged in terms of ED re-visits, hospital length of stay or mortality.

### Results in the context of the current literature

To our knowledge, no previous systematic reviews have investigated the effectiveness of HSCP teams in the ED. Our finding that CCTs in the ED reduced incidence of hospital admission and increased rates of community referrals is in-keeping with the findings of a previous systematic review [19] that reported a focus of ED care coordination on providing continuity of care post-discharge. On the other hand, while both Katz’s review [19] and another review [36] reported reductions in ED re-visits associated with CCTs, our review showed no impact of CCT interventions on ED re-visits at 28 days or 12 months. One reason for this difference might be that the included studies in our review focused on older adults only, who might have multiple reasons for re-attending the ED; clarifying the reason of the ED re-visit could help future studies to clarify this point. Considering our finding on unplanned hospitalisation rates, it is unclear from the included studies why a CCT intervention in the ED would result in higher rates of unplanned hospitalisations at 12 months when compared to routine care; once again, it is possible that multimorbidity might lead older adults to unplanned hospitalisations for reasons not related to the ED visit, but this needs further investigation, as only one study in our review explored this outcome.

We found that the CCT interventions were linked to improved patient and staff satisfaction, as well as improved quality of life, in two studies [32,33]. The results on patient satisfaction and quality of life are in line with previous studies investigating the role of HSCPs in the ED [10,12,36,37], and might be ascribed to a more personalised care and a longer interaction offered by allied health professionals. From the staff perspective, Innes et al [21] conducted a qualitative analysis of the impact of transdisciplinary care in the ED involving HSCPs and reported positive ED staff perceptions in terms of increased efficiency (more time available for medical staff) and quality of care, which are in line with our results. However, in our review patient and staff satisfaction were assessed differently in each study, limiting comparisons and thus our ability to conclude on the key factors that may influence satisfaction.

### Strengths and weaknesses of the review

Our review is the first to synthesise the totality of evidence on the effectiveness of team-based HSCP interventions in the ED. We used robust and transparent methods to identify, select, appraise and synthesise the study findings and we adhered to the standardised reporting guidelines to ensure rigour in the conduct and reporting of the research. Our findings indicate some level of effectiveness of HSCP interventions in the ED; however, the evidence is limited given the small number of available studies and the heterogeneity in study designs, index complaints and outcomes. The studies have important and significant methodologic limitations leading to unclear or high risks of bias for outcomes, and thus limit our ability to draw firm conclusions on effectiveness. The included studies were all conducted in Australia: This introduces a geographical bias limiting the generalisability to healthcare systems with different procedures for allied health professionals in the ED (i.e., CCT teams are routine ED care in Australia but may not be in other countries). Furthermore, the included studies focused on older patient groups, limiting generalisability of our findings to the entire ED population. Also, none of the studies included a dietician to screen for malnutrition, but this professional figure deserves investigation as there is emerging evidence to support their role as part of the HSCP team in the ED [38]. Importantly, none of the included studies presented findings on cost-effectiveness. However, analyses of cost-effectiveness of quality improvement strategies in the ED are limited in the literature [36], and in their review of the impact of ED models of care, Wylie et al. [36] concluded that having allied health professionals in the ED can have cost benefits due to improved clinical outcomes and reduced adverse effects, however those benefits have not been quantified systematically. Cost-effectiveness is a key factor in deciding the implementation of quality improvements strategies, and thus further investigation of this outcome is needed. A cost-effectiveness analysis from the perspective of the health care system should be conducted that considers the most optimal types of staffing resources (e.g., number of professionals, operational hours) in light of care and financial pressures. Lastly, we did not carry out a manual search of the grey literature which we acknowledge might introduce a potential publication bias; however, our database search included publication types from the grey literature (e.g., technical reports, conference abstracts, government documents).

### Clinical implications and areas for further research

Identifying cost-effective solutions to enhance patient flow is key to addressing the growing demands faced by EDs worldwide, and improving ED staffing resources is a key strategy to optimise patient care [9,39]. Extending the scope of practice of individual HSCPs to the ED has demonstrated good potential to ensure timely and effective care [10,11,14]. Our review advances this evidence by indicating that HSCP teams in the ED can reduce hospital admissions and improve referral pathways for older patients, and enhance patient and staff satisfaction.

In our review, we selected ED length of stay as primary outcome because it is considered a key measure of patient flow and ED performance [40], but no studies evaluated this outcome. Future research on the impact of HSCP interventions that focuses on ED length of stay will add to the evidence base regarding the effectiveness of this model of care.

The studies included in our review focused on older patient populations with a diverse range of health conditions, and only one study [1] evaluated the effectiveness of the intervention for each specific condition assessed. Including patients with diverse index complaints enhances the external validity of the study but it also provides a lack of clarity on the specific target populations that may benefit the most from the implementation of HSCP team-based interventions in the ED. Future research focusing on specific patient groups (e.g., older fallers, adults with chronic conditions) will be pivotal to address such questions. In addition, future investigations should look at different age groups to clarify whether the effectiveness of ED-based allied health professionals can be generalised to the entire ED adult population or it is specifically important for older ED attendees.

A lack of robust evidence on ED-based HSCP interventions has been highlighted in other reviews [10–12,36], and our findings further highlight the need for randomised controlled studies to reach clearer conclusions on the effectiveness of this model of care. Such trials should adhere to the relevant standardised reporting guidelines to standardise the conduct and reporting of the study; novel trial designs, such as stepped-wedge RCTs [41], should be considered to better address the pragmatic constraints of EDs.

## Conclusions

In this systematic review, we found some evidence that HSCPs working in teams can contribute to enhanced quality of care in the ED in the form of reduced hospital admissions, as well as improved patient and staff satisfaction. However, the limited number of studies and the presence of methodological heterogeneity across these studies highlight the need for further investigations on the clinical and cost effectiveness of this model of care using robust study designs and methods.

## Supporting information

S1 Appendix PRISMA checklist. PRISMA Checklist for reporting of systematic reviews.
(DOCX)

S2 Appendix Study searches. Detailed search strategy in the online databases.
(DOCX)

S1 Table Study characteristics. Characteristics of included studies.
(DOCX)
