# Supplementary material for: Impact of early assessment and intervention by teams involving health and social care professionals in the emergency department: A systematic review
Source: PLoS One. 2019 Jul 31;14(7):e0220709. doi: 10.1371/journal.pone.0220709 (PMC6668840; doi:10.1371/journal.pone.0220709)
Supplement: S2 Appendix — Detailed search strategy in the online databases. (DOCX) [file pone.0220709.s002.docx]

**S2 Appendix**

**Search strategy by Database**

**CINAHL on EBSCO** (1937 to 1^st^ March 2018, updated 8^th^ April 2019)

| **Search** | **Query** | **Items found** |
| --- | --- | --- |
| #1 | (MM "Emergency Service, Hospital") OR ( emergency room or emergency unit* or emergency department ) OR ED | 66363 |
| #2 | Asses* | 803031 |
| #3 | intervention or treatment or therapy | 1667655 |
| #4 | #2 OR #3 | 2112839 |
| #5 | #1 AND #4 | 33068 |
| #6 | (MH "Multidisciplinary Care Team+") OR multidisciplinary OR care coordination OR integrated care OR collaborative care OR team based care OR interdisciplinary | 82944 |
| #7 | physiotherap* OR physical therap* OR occupational therap* OR social work* OR pharmac* OR ( speech and language therap* ) OR ( speech therapy or speech patholog* or speech language patholog* ) OR allied health | 401498 |
| #8 | #6 OR #7 | 472718 |
| #9 | #5 AND #8 | 3812 |
| #10 | #9 Narrow by SubjectAge: - adolescent: 13-18 years + all adult: 19+ years | 1668 |
| #11 | #10 Updated search 1^st^ March 2018 – 8^th^ April 2019 | 102 |
|  | Total records screened | 1770 |

**Cochrane Library** (1920 to 1^st^ March 2018, updated 8^th^ April 2019)

| **Search** | **Query** | **Items found** | |
| --- | --- | --- | --- |
| #1 | MeSH descriptor: [emergency service, hospital] major topic OR emergency unit:ti,ab,kw OR emergency room:ti,ab,kw OR emergency department:ti,ab,kw | 18142 | |
| #2 | asses*:ti,ab,kw | 324692 | |
| #3 | intervention:ti,ab,kw or therapy:ti,ab,kw or treatment:ti,ab,kw | 664123 | |
| #4 | #2 OR #3 | 760158 | |
| #5 | #1 AND #4 | 13568 | |
| #6 | MeSH descriptor: [Patient Care Team] explode all trees | 1809 | |
| #7 | multidisciplinary:ti,ab,kw or interdisciplinary:ti,ab,kw or care coordination:ti,ab,kw or integrated care:ti,ab,kw or collaborative care:ti,ab,kw or team based care:ti,ab,kw | 10774 | |
| #8 | physiotherap*:ti,ab,kw or physical therap*:ti,ab,kw or occupational therap*:ti,ab,kw or social work*:ti,ab,kw or pharmacc*:ti,ab,kw or "speech and language therapist":ti,ab,kw or speech patholog*:ti,ab,kw | 36839 | |
| #9 | #7 OR #8 | 46022 | |
| #10 | #5 AND #9 | 1003 | |
| #11 | #10 in Trials | 962 | |
| #12 | #11 Updated search 1^st^ March 2018 – 8^th^ April 2019 | | 16 |
|  | Total records screened | | 978 |

**MEDLINE** **on EBSCO** (1946 to 1^st^ March 2018, updated 8^th^ April 2019)

| **Search** | **Query** | **Items found** |
| --- | --- | --- |
| #1 | (MM "Emergency Service, Hospital") OR ( emergency room or emergency unit* or emergency department ) OR ED | 292173 |
| #2 | Asses* | 2747680 |
| #3 | intervention or treatment or therapy | 6963104 |
| #4 | #2 OR #3 | 8641526 |
| #5 | #1 AND #4 | 139886 |
| #6 | (MH "Patient Care Team+") OR multidisciplinary OR care coordination OR integrated care OR collaborative care OR team based care OR interdisciplinary | 21719 |
| #7 | physiotherap* OR physical therap* OR occupational therap* OR social work* OR pharmac* OR ( speech and language therap* ) OR ( speech therapy or speech patholog* or speech language patholog* ) OR allied health | 4228362 |
| #8 | #6 OR #7 | 4415350 |
| #9 | #5 AND #8 | 27164 |
| #10 | #9 Narrow by SubjectAge: - adolescent: 13-18 years + all adult: 19+ years | 8431 |
| #11 | #10 Updated search 1^st^ March 2018 – 8^th^ April 2019 | 564 |
|  | Total records screened | 8995 |

**Embase** (1947 to 2^nd^ March 2018, updated 8^th^ April 2019)

| **Search** | | **Query** | **Items found** | |
| --- | --- | --- | --- | --- |
| #1 | | 'emergency ward'/exp/mj OR 'emergency ward'/mj OR 'emergency room':ti,ab,kw OR 'emergency unit':ti,ab,kw OR 'emergency department':ti,ab,kw OR 'ed':ti,ab,kw | 186557 | |
| #2 | | 'asses*':ti,ab,kw | 3403176 | |
| #3 | | 'intervention':ti,ab,kw OR 'treatment':ti,ab,kw OR 'therapy':ti,ab,kw | 6709358 | |
| #4 | | #2 OR #3 | 8930338 | |
| #5 | | 'interdisciplinary':ti,ab,kw OR 'multidisciplinary':ti,ab,kw OR 'collaborative care':ti,ab,kw OR 'care coordination':ti,ab,kw OR 'integrated care':ti,ab,kw OR 'team based care':ti,ab,kw | 152061 | |
| #6 | | 'physiotherap*':ti,ab,kw OR 'physical therap*':ti,ab,kw OR 'occupational therap*':ti,ab,kw OR 'social work*':ti,ab,kw OR 'pharmac*':ti,ab,kw OR 'speech and language therap*':ti,ab,kw OR 'speech patholog*':ti,ab,kw | 1085232 | |
| #7 | | #1 AND #4 | 94451 | |
| #8 | | #5 OR #6 | 1219311 | |
| #9 | | #7 AND #8 | 9086 | |
| #10 | | #9 AND ([adult]/lim OR [aged]/lim OR [middle aged]/lim OR [very elderly]/lim OR [young adult]/lim) | 3445 | |
| #11 | #10 Updated search 1^st^ March 2018 – 8^th^ April 2019 | | | 500 |
|  | Total records screened | | | 3945 |
