# Supplementary material for: Impact of early assessment and intervention by teams involving health and social care professionals in the emergency department: A systematic review
Source: PLoS One. 2019 Jul 31;14(7):e0220709. doi: 10.1371/journal.pone.0220709 (PMC6668840; doi:10.1371/journal.pone.0220709)
Supplement: S1 Table — Characteristics of included studies. (DOCX) [file pone.0220709.s003.docx]

**S1 Table**

**Characteristics of included studies (based on TIDieR guidelines)**

| **Reference**  **Country**  **Design** | **Population**  **Inclusion criteria**  **Sample size (N)**  **Group size (n), age (mean, SD), sex (Percentage female)** | **Health condition** | **Team** | **Assessment/Intervention** | **Comparison(s)** | **Outcome(s)** | **Results** |
| --- | --- | --- | --- | --- | --- | --- | --- |
| **Arendts et al. (2012)**  (1)  Australia  nRCT | Included:  Patients aged 65+ with one or more of 10 medical complaints (infectious, musculoskeletal, cardiovascular, neurological)  N = 5265  Intervention  - n = 3165  - mean age 80 (SD = 8)  - 56% female  Control  - n = 2100  - mean age79 (SD =8)  - 54% female | One or more conditions across 4 domains:  - Infectious;  - Musculoskeletal  - Cardiovascular  - Neurological | Care Coordination team (CCT)  Composition:  - OT;  - PT;  - MSW.  Co-opted physician, nurse and SLT. | Formal comprehensive functional assessment by at least one CCT member, then initiation of services:  - discharge needs,  - falls risk,  - activities of daily living (ADLs),  - cognition.  Assessment incorporated into final medical decision (ED physician) for discharge or hospital admission.  Services to address any needs identified on assessment not otherwise specified  No follow-up | Individuals eligible for CCT assessment but undergoing medical assessment only | Proportion of hospital admissions from the ED | Unadjusted 2.4% reduction in hospital admissions for intervention vs. control  Adjusted comparisons (propensity score matching) reduced hospital admissions for musculoskeletal complaints and angina |
| **Arendts et al. (2013)**  (30)  Australia  nRCT | Included:  - Aged 65+  - Community-dwelling  - Dischargeable from ED  - Not requiring resuscitation or emergency medical care  N = 2196  Intervention (patients with functional risks):  - n = 1098  - mean age = 78 (8)  - 56% female  Control:  - n = 1098  - mean age = 77 (8)  - 56% female | Various complaints including:  - falls with no/minor injury,  - ischaemic chest pain,  - atraumatic musculoskeletal pain,  - dizziness | Care Coordination team (CCT):  - OT;  - PT;  - SW.  Co-opted physician, nurse and SLT. | Formal comprehensive functional assessment by at least one CCT member, then initiation of services:  - discharge needs,  - falls risk,  - ADLs,  - cognition.  Where necessary,  CCT care included subsequent referral to post-discharge services to address any medical, allied health  or social needs encountered in the CCT assessment  Follow-up at 28 days and one year post-discharge | Older patients with no risks identified on this screening process, thus deemed low risk for discharge if their clinical condition allowed, and not undergoing comprehensive assessment unless specifically referred for such by medical staff.  Intervention group matched in a 1:1 ratio to controls based on: age in 5-year age band, sex, weekday versus weekend arrival, time of arrival in 8 h time band, ICD-10 principal diagnosis using only the first three alphanumeric characters of the code and triage urgency | 1. Unplanned ED re-visit within 28 days  2. 28 days and one-year mortality  3. morbidity (unplanned hospitalisations) at 1 year | 1. 3% absolute difference in 28-day unplanned ED re-visits rates between cases (n = 196 [17.9%]) and controls (n = 162 [14.8%]); c2[1] = 3.86, p = .05.  2. No differences in mortality at 28 days (1.4% vs. 1.3%, p = .85) or one year (10.7% vs. 10.2%, p = .66)  3. Higher rate of unplanned hospitalisations in intervention (43.4%) vs. control (29.5%), p <.001. |
| **Arendts et al. (2013)**  (31)  Australia  nRCT | Included:  - Aged 65+  - Being admitted to inpatient bed from the ED (excluding ED observation unit and ICU)  - Primary indicator for admission was one of six diagnoses (see health condition)  N = 3672  Intervention:  - n = 2121  - mean age 80 (SD = 80)  - 53% female  Control:  - n = 1451  - mean age 80 (SD = 80)  - 55% female | Six index diagnoses:  - cerebrovascular insufficiency,  - fractured neck of femur,  - cardiac failure,  - myocardial ischaemia (IDH),  - exacerbation of chronic airways disease (CAL);  - respiratory infection (LRTI). | Care coordination team (CCT):  - OT;  - PT;  - SW.  Co-opted nurse and other AHS (SLT). | Comprehensive assessment by at least on CCT member to determine functional needs and potential barriers to subsequent discharge from hospital.  Assessment results included in patient notes and verbally communicated with inpatient units  No follow up | Eligible patients who did not receive CCT assessment (type of care received not specified) | Hospital length of stay (hours) | No significant differences in hospital length of stay between intervention and control, either in adjusted or unadjusted analyses. |
| **Corbett et al. (2005)**  (32)  Australia  RMS | Included:  - aged 65+  - expected to be discharged in the community  N = 175400  Pre-intervention:  - n = 40510  One year post-intervention (T1):  - n = 42196  Two years post-intervention (T2):  - n = 44456  Three years post-intervention (T3):  - n = 48238 | Not specified | Care Coordination Team (CCT):  - OT  - Nurse  - PT  - SLT  - MSW | Establishing a case management for coordination and provision of services and programs for patients with complex care needs upon discharge from the ED back into the community.  Not specified if formal or informal  The CC team provided services, however, their primary role was coordination (not otherwise specified)  No follow-up | Patients aged 65+ visiting the ED and being admitted to wards in the year pre-intervention | 1. Percentage of admissions to inpatient wards one (T1), two (T2) and three years (T3) after introduction of CCT  2. Health-related quality of life of patients undergoing CCT assessment, administered pre-assessment and 1-month follow-up (N = 11).  3. Patient and staff satisfaction (assessed via focus group post-intervention only) | 1. Significant decrease in hospital admissions from pre-intervention (20.16%) to T1 (18.10%), T2 (18.39%), and T3 (18.03%), p < .005.  2. Significant improvements for five of six dimensions  3. Positive perceptions by staff, moderate-positive perceptions by patients |
| **Moss et al. (2002)**  (33)  Australia  RMS | Included:  - Frail adults 65+;  - Living alone;  - Frequent ED users;  - requiring help with ADLs;  - having complex medical problems;  - not eligible for home hospital;  - homeless;  - requiring complex discharge planning;  - with drug or alcohol problems  N = 87040  Intervention (year after CCT introduction) n = 43430, of which 2532 (5.8%) assessed by the CCT  Control (year before CCT introduction) n = 43610  not provided.  No patient demographic information provided. | Various complaints including:  - respiratory;  - cardiac;  - musculoskeletal;  - neurological;  - abdominal;  - infections;  - wounds;  - confusion and dementia;  - psychiatric;  - drug and alcohol;  - mixed medical/social;  - purely social;  - other medical. | Care Coordination Team (CCT):  - OT  - Nurse  - MSW | Comprehensive discharge risk assessment involving documentation of expected discharge date and destination, existing services and supports, and indication for referral to internal and external health professionals.  In addition, information and/or education was provided to patient and family.  The CCT may undertake home visits.  One-year follow-up | Patients visiting the ED and being admitted to wards in the year pre-intervention (no matching of participants) | 1. Rate of hospital admissions  2. ED re-visits  3. Patient/carer satisfaction (n = 40 completed telephone survey one week after discharge)  4. Staff satisfaction (68 ED staff completed mail survey six months after CCT introduction)  5. Community service providers satisfaction (16 completed mail survey one year after CCT introduction)  6. Rates and types of discharges from ED | 1. Significant reduction in hospital admissions post-CCT introduction (13 420 patients,  30.9% [95% CI, 30.5–31.3] vs. 14 217 patients, 32.6% [95% CI, 32.2–33.0]; p < .001)  2. No significant differences in ED re-visits at 12 months (3744 post vs. 3856 pre; p = .28)  3. 100% of patients/carers rated the CCT services as good or very good.  Respondents reported that the CCT assisted in the provision of safe and effective discharge, provided high quality service and could be recommended.  4. Over 92% of ED staff rated the CCT as providing quality patient care, having a positive impact on patient discharge, being easily accessible, increasing staff morale, and worth recommending to other EDs.  5. CCT rated as easily accessible (68%), having a positive impact on patients’ outcomes (75%), and worth recommending to other EDs (87.5%)  6. 85.1% of 2532 seen by the CCT discharged home, 15.1% admitted, 3.2% transferred. |
| **Waldron et al. (2011)**  (34)  Australia  CBA | Included:  - Aged 65+ (or 45+ if aboriginal or torres strait islanders)  - Community-dwelling  - Discharged directly from ED  N = 313  Intervention (after):  - n = 136  - mean age = 76.54 (SD = 8.07)  - 70.6% female  Control (before):  - n = 177  - mean age = 75.86 (SD = 7.84)  - 69.5% female | Falls | Care coordination team (CCT):   - OT - PT - MSW | CCT trained to use a stratified referral pathway to identify the risk of falls of older patients and refer them to multifactorial or single interventions in the community.  Not all patients seen by CCT.  No follow-up. | Patients presenting to the ED following a fall before the implementation of referral pathway | 1. proportion of patients reviewed by the CCT after introduction of referral pathway;  2. proportion of patients referred for multi-factorial interventions  3. proportion referred for single interventions  4.Quality of care (15-items composite score) | 1. Increased number of patients reviewed by CCT after, 62% to 89% (p < .001)  2. Increased number of patients referred to guideline care, from 3.4% to 20.6% (p < .001  3. Trend towards increased number of referrals to single interventions, from 13% to 21.3% (p = .05)  4. 75.3% improvement in quality of care index  Increase in CCT staffing during study but not associated with percentage of referrals to multifactorial interventions |
| \| *C2 = Chi-square test; CBA = Controlled Before-After study; CCT = Care Coordination Team; CP = Clinical Pharmacist; ED = Emergency Department; ICD-9-CM = International Classification of Diseases, Ninth Revision, Clinical Modification; MSW = Medical Social Worker; N = Sample size; n = Group size; nRCT = nonrandomised Controlled Trial; OT = Occupational Therapist; PC = Primary Care; PT = Physiotherapist; RCT = Randomised Controlled Trial; RMS = Repeated Measures Study; SD = Standard Deviation; SLT = Speech and Language Therapist; UC = Urgent Care.* \| \| --- \| | | | | | | | |
